# Specific and sensitive quantitative RT-PCR of miRNAs with DNA primers

**DOI:** 10.1186/1472-6750-11-70

**Published:** 2011-06-25

**Authors:** Ingrid Balcells, Susanna Cirera, Peter K Busk

**Affiliations:** 1 Departament de Ciència Animal i dels Aliments, Universitat Autònoma de Barcelona, 08193 Bellaterra, Spain; 2 Department of Animal and Veterinary Basic Sciences, University of Copenhagen, Copenhagen, Denmark; 3 Department of Biotechnology, Chemistry and Environmental Engineering, Aalborg University, Lautrupvang 15, 2750 Ballerup, Denmark

## Abstract

**Background:**

MicroRNAs are important regulators of gene expression at the post-transcriptional level and play an important role in many biological processes. Due to the important biological role it is of great interest to quantitatively determine their expression level in different biological settings.

**Results:**

We describe a PCR method for quantification of microRNAs based on a single reverse transcription reaction for all microRNAs combined with real-time PCR with two, microRNA-specific DNA primers. Primer annealing temperatures were optimized by adding a DNA tail to the primers and could be designed with a success rate of 94%. The method was able to quantify synthetic templates over eight orders of magnitude and readily discriminated between microRNAs with single nucleotide differences. Importantly, PCR with DNA primers yielded significantly higher amplification efficiencies of biological samples than a similar method based on locked nucleic acids-spiked primers, which is in agreement with the observation that locked nucleic acid interferes with efficient amplification of short templates. The higher amplification efficiency of DNA primers translates into higher sensitivity and precision in microRNA quantification.

**Conclusions:**

MiR-specific quantitative RT-PCR with DNA primers is a highly specific, sensitive and accurate method for microRNA quantification.

## Background

MicroRNAs (miRNAs) are small non-coding RNAs that are important regulators of biological processes in animals and plants. MiRNAs regulate gene expression at the posttranscriptional level by binding to mRNAs and either inhibit translation or modify the stability of the mRNA. Due to the important biological role of miRNAs it is of great interest to study their expression level in the cells. Furthermore, miRNAs have been associated with cancer and other diseases [1] and miRNA expression can help in the diagnosis and prognostic of human disease [2,3]. The discovery of miRNAs in blood and their surprisingly high stability holds great promise for diagnosis of human disease with miRNAs as biomarkers [4]. Several studies have shown that the amount of individual miRNAs in blood is affected by human disease and that the level of specific miRNAs can be used as a diagnostic tool (for examples see [5-9]).

The three methods most frequently used for detection of miRNAs are high-throughput sequencing, microarrays and reverse transcription quantitative PCR (RT qPCR). The latter method is used independently and for validating data obtained from high-throughput sequencing and microarrays. It is challenging to design PCR primers for miRNAs as the typical miRNA is only 22 bases long, which is about the same size as a conventional PCR primer. Several methods have been developed to overcome this problem. Chen and coworkers [10] developed stem-loop RT-PCR where reverse transcription is done at low temperature with a specially designed loop-primer followed by PCR with one specific primer and a universal primer. The PCR product is detected with a TaqMan probe. Although the method requires a specific RT primer for each miRNA, this method can be performed as multiplex so that one RT reaction can be used as template for several qPCR reactions [11]. Unfortunately, stem-loop RT-PCR does not allow the user to control the specificity of the reaction by melting curve analysis and the TaqMan probe does not contribute to specificity as the probe binds to the part of the cDNA sequence that originates from the RT primer. Thus, if the RT primer binds to another sequence than the miRNA of interest, this will lead to incorporation of the binding site of the TaqMan probe and this unspecific amplicon will be indistinguishable from the desired PCR product.

The recently published method based on circularization of the miRNA also depends on a specific primer for reverse transcription [12] and may be difficult to adapt to multiplexing. Furthermore, circularization by RNA ligase is sensitive to sequence bias [13].

Another way to perform miRNA qPCR is to add a poly(A) tail to the miRNA and use a tagged poly(T) primer for reverse transcription [14]. Subsequently, PCR is performed with a miRNA-specific primer and a universal primer. This method is very convenient when the amount of sample is limiting, which is often the case for samples such as biopsies and microdissected samples, and when miRNA concentrations are low such as in blood, because it only requires a single RT reaction to generate a template for detection of all miRNAs. However, as only one specific primer is used for PCR there is little degree of freedom in primer design and specificity could be an issue. Especially the discrimination between closely related miRNAs that differ by only one or a few nucleotides can be difficult using this method.

The method called Universal RT microRNA PCR combines the benefits of a universal RT reaction with the specificity of two miRNA-specific PCR primers [15]. The PCR product is detected with the intercalating dye SYBR-Green that allows the control of unwanted PCR products by melting curve analysis. The method relies on poly(A) tailing of the miRNAs followed by reverse transcription with a tagged poly(T) primer. PCR is performed with two specific primers that are spiked with Locked Nucleic Acid (LNA) to increase the Tm and the specificity. Although the PCR reactions are specific and discriminate well between closely related miRNAs, they often exhibit a low amplification efficiency which is a common cause of inaccurate quantification. This is in agreement with the observation that sequences containing LNA are poor templates for most DNA polymerases [16].

In the present study we describe that qPCR with two miRNA-specific DNA primers leads to higher amplification efficiency than qPCR with LNA-spiked primers. In addition, this method has all the benefits regarding freedom of primer design and specificity of the LNA-based method. Optimization of primer Tm and high specificity of the PCR reaction is achieved by adding a tail to each of the PCR primers.

## Results

**Figure 1:**
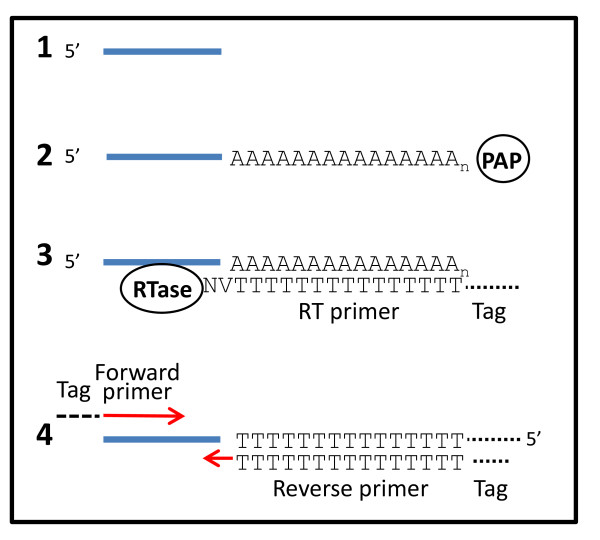
**Flow scheme of miR-specific qPCR**. **1. **Start with purified RNA containing miRNA. **2. **Add poly(A) tail with poly(A) polymerase (PAP). **3. **cDNA synthesis with reverse transcriptase (RTase) and an anchored poly(T) primer with a 5' tag. **4. **PCR with two tagged primers.

**Figure 2:**
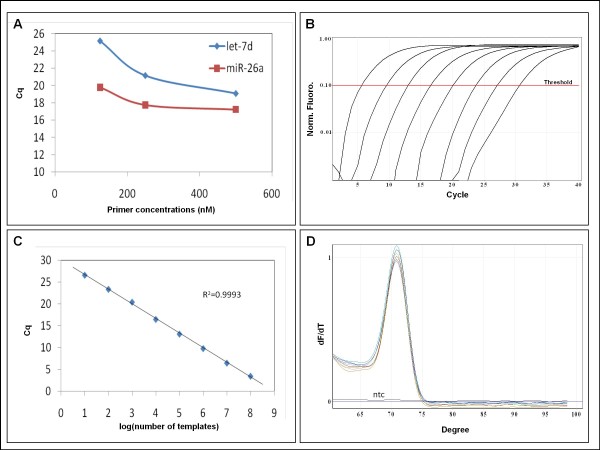
**MiR-specific qPCR on synthetic templates with DNA primers**. **A **The effect of primer concentration on Cq value of *ssc-let-7d *and *ssc-miR-26a *miR-specific qPCR assays. Real-time PCR assays were performed in parallel at three different concentrations (125, 250 and 500 nM) of the forward and of the reverse primers. **B **Amplification curves of an eight log_10 _dilution series of a synthetic *ssc-let-7d *template in the *ssc-let-7d *miR-specific qPCR assays. All samples contained a final concentration of 0.2 ng/μl salmon sperm DNA. **C **Extrapolation of Cq as function of the log_10 _of the number of templates for the same experiment as in B was a straight line (R^2 ^= 0.9993) with slope of -3.341 (PCR efficiency = 99%) over eight log_10 _dilution of the template. **D **Melting curve analysis of the same experiment. No template control is labeled ntc. Melting curve analysis was performed from 60°C to 99°C.

MiR-specific qPCR of miRNAs combines the benefits of a universal RT reaction with the specificity of two miR-specific primers for qPCR (Figure 1). We designed miR-specific DNA primers (Table 1) and tested them at different concentrations in real-time PCR of synthetic miR templates in a background of salmon sperm DNA. A final concentration of 250 nM of each primer was found to be optimal for qPCR (Figure 2A). This primer concentration gave significantly lower Cq values than 125 nM primer whereas 500 nM primer did not reduce the Cq values further.

**Table 1:** MiRNAs, PCR primers and synthetic templates

miRNA	Sequence	Forward primer	Reverse primer	Synthetic template
let-7a	UGAGGUAGUAGGUUGUAUAGUU	GCAGTGAGGTAGTAGGTTGT	GGTCCAGTTTTTTTTTTTTTTTAACTATAC	CAGGTCCAGTTTTTTTTTTTTTTTAACTATACAACCTACTACCTCA
let-7d	AGAGGUAGUAGGUUGCAUAGUU	AGAGAGGTAGTAGGTTGCAT	AGGTCCAGTTTTTTTTTTTTTTTAACT	CAGGTCCAGTTTTTTTTTTTTTTTAACTATGCAACCTACTACCTCT
miR-20a	UAAAGUGCUUAUAGUGCAGGUAG	ACAGTAAAGTGCTTATAGTGCA	GTCCAGTTTTTTTTTTTTTTTCTACCT	CAGGTCCAGTTTTTTTTTTTTTTTCTACCTGCACTATAAGCACTTTA
miR-21	UAGCUUAUCAGACUGAUGUUGA	TCAGTAGCTTATCAGACTGATG	CGTCCAGTTTTTTTTTTTTTTTCAAC	CAGGTCCAGTTTTTTTTTTTTTTTCAACATCAGTCTGATAAGCTA
miR-23a	AUCACAUUGCCAGGGAUUUCCA	CATCACATTGCCAGGGAT	CGTCCAGTTTTTTTTTTTTTTTGGAA	CAGGTCCAGTTTTTTTTTTTTTTTGGAAATCCCTGGCAATGTGAT
miR-23b	AUCACAUUGCCAGGGAUUACCAC	same as for miR-23a	TCCAGTTTTTTTTTTTTTTTGTGGTA	CAGGTCCAGTTTTTTTTTTTTTTTGTGGTAATCCCTGGCAATGTGAT
miR-25	CAUUGCACUUGUCUCGGUCUGA	CATTGCACTTGTCTCGGT	GGTCCAGTTTTTTTTTTTTTTTCAGA	
miR-26a	UUCAAGUAAUCCAGGAUAGGCU	CGAGTTCAAGTAATCCAGGA	CCAGTTTTTTTTTTTTTTTAGCCTATC	CAGGTCCAGTTTTTTTTTTTTTTTAGCCTATCCTGGATTACTTGAA
miR-27a	UUCACAGUGGCUAAGUUCCGC	CAGTTCACAGTGGCTAAGA	CAGTTTTTTTTTTTTTTTGCGGAA	CAGGTCCAGTTTTTTTTTTTTTTTGCGGAACTTAGCCACTGTGAA
miR-101a	UACAGUACUGUGAUAACUGAA	CGCAGTACAGTACTGTGATAAC	AGGTCCAGTTTTTTTTTTTTTTTCAG	CAGGTCCAGTTTTTTTTTTTTTTTCAGTTATCACAGTACTGTA
miR-103	AGCAGCAUUGUACAGGGCUAUGA	AGAGCAGCATTGTACAGG	GGTCCAGTTTTTTTTTTTTTTTCATAG	
miR-122	UGGAGUGUGACAAUGGUGUUUGU	ACAGTGGAGTGTGACAATG	TCCAGTTTTTTTTTTTTTTTCAAACAC	CAGGTCCAGTTTTTTTTTTTTTTTACAAACACCATTGTCACACTCCA
miR-125b	UCCCUGAGACCCUAACUUGUGA	CAGTCCCTGAGACCCTA	GTCCAGTTTTTTTTTTTTTTTCACAA	CAGGTCCAGTTTTTTTTTTTTTTTCACAAGTTAGGGTCTCAGGGA
miR-139b-5p	UCUACAGUGCACGUGUCUCCAGU	TCTACAGTGCACGTGTCT	GTCCAGTTTTTTTTTTTTTTTACTGGA	CAGGTCCAGTTTTTTTTTTTTTTTACTGGAGACACGTGCACTGTAGA
miR-150	UCUCCCAACCCUUGUACCAGUG	GTCTCCCAACCCTTGTAC	GTCCAGTTTTTTTTTTTTTTTCACTG	CAGGTCCAGTTTTTTTTTTTTTTTCACTGGTACAAGGGTTGGGAGA
miR-199b-3p	UACAGUAGUCUGCACAUUGGUU	CAGTACAGTAGTCTGCACAT	GTCCAGTTTTTTTTTTTTTTTAACCAA	CAGGTCCAGTTTTTTTTTTTTTTTAACCAATGTGCAGACTACTGTA
miR-200b	UAAUACUGCCUGGUAAUGAUGA	ACAGTAATACTGCCTGGTAATG	GGTCCAGTTTTTTTTTTTTTTTCATC	CAGGTCCAGTTTTTTTTTTTTTTTCATCATTACCAGGCAGTATTA
miR-200c	UAAUACUGCCGGGUAAUGAUGGA	AGTAATACTGCCGGGTAATG	GTCCAGTTTTTTTTTTTTTTTCCATC	CAGGTCCAGTTTTTTTTTTTTTTTCCATCATTACCCGGCAGTATTA

The PCR reactions were linear over a range of eight log_10 _of synthetic template (Figure 2B and 2C), produced one peak in melting curve analysis (Figure 2D) and exhibited a good correlation between Cq and template concentration (Figure 2C).

**Figure 3:**
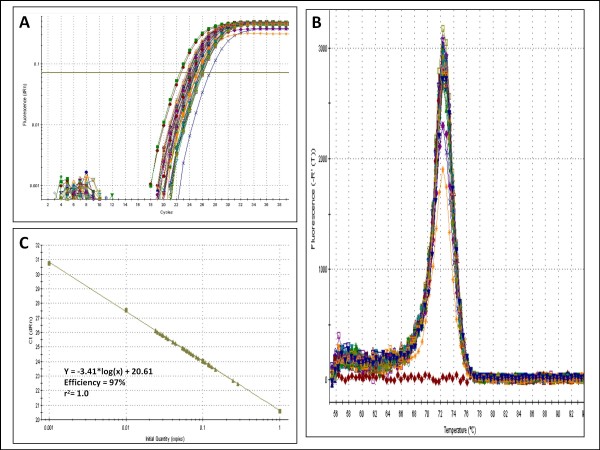
**MiR-specific qPCR on biological samples with DNA primers**. **A **Amplification curves of 40 uterus samples with the *ssc-miR-150 *miR-specific qPCR assay. **B **Melting curve analysis of the same experiment. Melting curve analysis was performed from 55°C to 95°C. C Extrapolation of Cq as function of the log_10 _of the number of templates for the same experiment as in A was a straight line (R^2 ^= 1.0) with a slope of -3.406 (PCR efficiency = 97%) over 4 log_10 _dilution of a pool that includes all samples included in the study.

Amplification of miRNAs from biological samples yielded similar amplification curves as for synthetic templates (Figure 3A) and melting curve analysis indicated the presence of only one amplicon (Figure 3B). In addition, there was a good correlation between Cq and template concentration over four log_10 _dilutions when biological samples were used (R^2 ^≥ 0.98) (Figure 3C).

To test the hypothesis that LNA can inhibit PCR amplification by decreasing the amplification efficiency we compared the efficiency of amplification of 18 miRNAs from porcine uterus with commercially available LNA-spiked primers sets from Exiqon (Denmark) and with DNA primers without LNA. With LNA-spiked primers, amplification efficiencies ranged from 79 to 95% for 17 of the 18 assays. The last assay (let-7d) had an apparent efficiency of 85% but more than one peak appeared in the melting curve analysis of the PCR product (data not shown). This indicates that the assay is unspecific and it was excluded from the analysis of assay efficiency (Table 2). Amplification efficiencies with DNA primers ranged from 84 to 102% (Table 2) and were significantly higher than with LNA-spiked primers (*P*-value < 0.001). On average, the PCR reactions with DNA primers yielded 5.0% higher efficiency than LNA-spiked primers corresponding to a 2.4 fold higher sensitivity after 30 cycles of PCR. Melting curve analysis of the let-7d assay with DNA primers only yielded one peak corroborating that this assay was specific (Figure 2D).

**Table 2:** Efficiency of miR-specific qPCR assays with LNA-spiked and DNA primers on pig uterus total RNA

Target	Efficiency LNA primers	Efficiency DNA primers	Difference
let-7a	82%	89%	6.9%
miR-101a	85%	90%	4.9%
miR-103	93%	94%	1.6%
miR-122	95%	95%	-0.1%
miR-125b	89%	94%	4.5%
miR-139b-5p	79%	86%	6.4%
miR-150	84%	97%	12.6%
miR-199b-3p	80%	87%	7.1%
miR-20a	88%	86%	-2.0%
miR-200b	80%	94%	13.6%
miR-200c	83%	84%	0.2%
miR-21	91%	92%	1.1%
miR-23a	79%	93%	14.1%
miR-23b	81%	87%	6.2%
miR-25	84%	91%	6.7%
miR-26a	88%	96%	8.3%
miR-27a	86%	85%	-1.1%
let-7d	not specific	102%	

**Average**	**85%**	**90%**	**5.4%**

**Figure 4:**
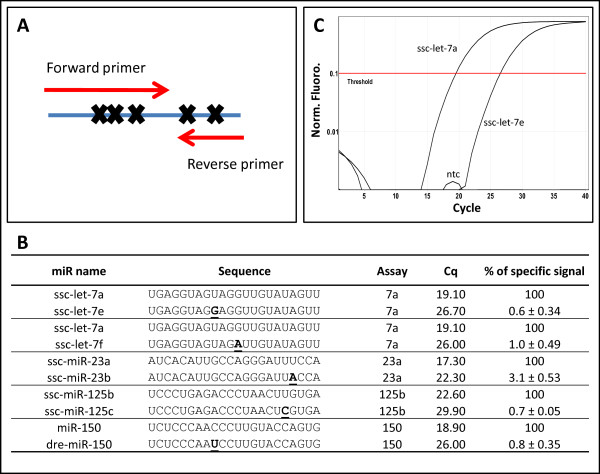
**Discrimination between miRNAs with single nucleotide differences**. **A **Position of the single nucleotide mismatches relative to the PCR primers for the *ssc-let-7a, ssc-miR-23a, ssc-miR-125b *and *ssc-miR-150 *qPCR assays. The *ssc-miR-23b *sequence used for mismatch discrimination was taken from miRBase and is different from the *ssc-miR-23b *sequence found in uterus and used for designing the *ssc-miR-23b *qPCR primers (Table 1). **B **Discrimination between closely related miRNA templates for miR-specific qPCR assays with DNA primers. Mismatches in the miRNA compared to the PCR primers are underlined. The data represents the results of three to four measurements. **C **Amplification curves of *ssc-let-7a *and *ssc-let-7e *synthetic template in the *ssc-let-7a *miR-specific qPCR assays. All samples including the no template control (ntc) contained a final concentration of 0.2 ng/μl salmon sperm DNA.

The ability of DNA primers to distinguish between miRNAs with a single base difference was tested for three cases where the one base difference was in the part of the miRNA sequence used for forward primer design and two cases where the difference was in the sequence used for reverse primer design (Figure 4A). On average, qPCR of the specific template gave almost 100-fold higher signal than amplification of the template with a single base difference (Figure 4B). For example, amplification of let-7a with the let-7a assay gave a Cq that was 7.6 cycles lower than amplification of the same amount of let-7e with the let-7a assay corresponding to a difference of 170 fold in favor of the intended template compared to the single base mismatch (Figure 4C).

**Figure 5:**
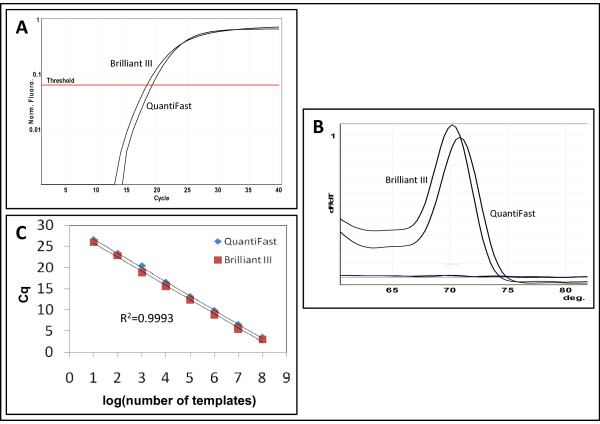
**MiR-specific qPCR in different qPCR master mixes**. **A **Comparison of amplification curves of a synthetic *ssc-let-7d *template in the *ssc-let-7d *miR-specific qPCR assay in QuantiFast and in Brilliant III qPCR Master mixes. **B **Melting curve analysis of the same experiment. No template control is labeled ntc. Melting curve analysis was performed from 60°C to 99°C. No change in fluorescence (dF/dT = 0) was observed above 80°C and this part of the curves was omitted from the figure. **C **Extrapolation of Cq as function of the log_10 _of the number of templates for the same experiment as in A was a straight line (R^2 ^indicated on figure) and for both master mixes the PCR efficiency was 99% as calculated from the slope of the regression line.

To investigate the effect of different PCR master mixes on the performance of miR-specific qPCR with DNA primers we compared the amplification of synthetic templates with the QuantiFast SYBR Green PCR master mix (Qiagen, Germany) and the Brilliant III Ultra-Fast QPCR Master Mix (Agilent, USA). There was no difference in amplification efficiency (*P*-value = 0.69) for the five assays tested (let-7d, miR-20a, miR-21, miR-26a and miR-150) between the two master mixes and all the assays gave one peak in melting curve analysis and were comparable over eight log_10 _of template concentration (Figure 5). The different Tm (peak of the melting curve) in the two master mixes may probably be attributed to different composition of the buffers.

MiR-specific qPCR of let-7a, miR-21, miR-23a and miR-150 with DNA primers on RNA from six different pig tissues showed expression levels from 8 copies per pg total RNA up to almost 2000 copies per pg total RNA (Table 3). Expression of let-7a was remarkably stable with differences below 5 fold between the six tissues. Regardless of the level of expression (Cqs from 16 to 23) and the type of tissue, the assays yielded products with one peak in melting curve analysis as expected for specific PCR amplifications (data not shown). The same expression profile of the four miRs in the same six samples (*P*-values > 0.05) was obtained with LNA primers but the Cq values were one cycle higher on average (data not shown).

**Table 3:** Expression profiling of four miRNAs in pig tissues measured by miR-specific qPCR with DNA primers

miRNA	brain	heart	liver	lung	thymus	ovary	Cq(min)	Cq(max)
**let-7a**	120	87	27	120	34	98	16.2	18.8
**miR-21**	88	190	36	900	340	1800	15.9	20.7
**miR-23a**	15	42	8	100	11	33	16.2	20.4
**miR-150**	39	22	19	140	270	21	18.6	23.4

## Discussion

MiR-specific qPCR is a relatively new method that holds great promise. The use of two miR-specific primers makes the method as specific as stem-loop RT-PCR and the reverse transcription is performed with a universal primer compatible with all qPCR primer pairs and is therefore optimal for analysis of small RNA samples and for high-throughput screening [15]. Furthermore, detection with intercalating dye allows characterization of the PCR product by melting curve analysis. MiRNA PCR may produce unwanted side products that can only be detected by melting curve analysis.

Commercially available primers for miR-specific qPCR are spiked with LNA (http://www.exiqon.com). In the present study we found that qPCR reactions with LNA-spiked primers had a tendency to exhibit low amplification efficiencies, which makes accurate quantification more difficult [17]. Although several algorithms that account for amplification efficiency are available to calculate the original template concentration from real-time PCR data [18-21] low amplification efficiency is a sign that the amplification reaction is suboptimal and will in all cases lead to lower sensitivity of the PCR reaction [22]. However, we found that DNA primers can be successfully used for miR-specific qPCR and that the use of DNA gives significantly higher amplification efficiencies than LNA-spiked primers. Low Tm is often a problem in case of the short primers designed for a miRNA template. This issue can be solved by spiking LNA into the sequence to increase the Tm [23]. However, the same can be achieved by adding an artificial sequence to the 5' end of the primer as done for the stem-loop RT-PCR method [10]. In the present report we optimized forward primer Tm to 59°C by adding an artificial sequence at the 5' end and found that these primers performed well in miR-specific qPCR. The reverse primer for miR-specific PCR is constructed with a short, specific sequence that varies from 4-8 bases at the 3' end followed by a 15 bases long thymidine stretch as in the RT primer and finally, a 5' end tail (tag) that can be varied in length to optimize the Tm [15]. Strictly speaking the primer is not specific as only the last 4 - 8 bases in the 3' end are complementary to the miRNA. However, this short sequence combined with the thymidine stretch is sufficient to confer high specificity to the PCR reaction. E.g. templates without a polyA tail or premiRs that extend the miR at the 3' end are not amplified [15]. It was reported that it is necessary to spike an LNA into the reverse primer to avoid aberrant amplification products but this effect was only demonstrated for primers with very high Tm (67 - 68°C ) [15]. We found that when the Tm of the reverse primer is optimized to 59°C, which is the optimal Tm for the forward primer, the LNA is no longer crucial for successful PCR.

A possible explanation of the lower amplification efficiency with LNA-spiked primers is that for short targets such as miRNAs the primers that are incorporated into the template during amplification will lead to a high proportion of LNA in the template that will decrease the efficiency of subsequent PCR cycles. This possibility is supported by differences between the solution structure of a DNA:LNA helix and the structure of double-stranded DNA [24] and that nucleotide incorporation opposite to an LNA base may be difficult for some polymerases [16]. A second possibility is that the LNA-spiked primers may be more prone to form secondary structures that will lower the efficient primer concentration available to hybridize to the template. Stem-loop RT PCR is performed with DNA primers [10] and should therefore have the same efficiency as miR-specific qPCR with DNA primers provided that the detection method does not influence efficiency. Measurement of the efficiency of 87 stem-loop RT PCR assays gave an average efficiency of 94% ± 0.09 [25]. As expected this efficiency is not significantly different from the average efficiency (91% ± 0.05) for the 18 miR-specific qPCR assays with DNA primers reported in the present study (*P*-value = 0.17, Student's T-test) but it is higher than the average efficiency (85% ± 0.05) for the 17 miR-specific qPCR assays with LNA-spiked primers reported in the present study (*P*-value = 0.0001, Student's T-test). It therefore seems that DNA primers give higher amplification efficiency of miRNA templates than LNA-spiked primers independently of whether intercalating dye or TaqMan probes are used for detection.

The lower dissociation rates of double-stranded DNA containing LNA bases [26] suggest that PCR with LNA-spiked primers requires longer denaturation times. However, the recommended protocol (http://www.exiqon.com) has a denaturation time of 30 seconds which should be more than sufficient.

The use of two specific primers for each miRNA allows for design of several different primer sets. E.g, for a 22 bases sequence the forward primer can be from 15-18 bases long and the reverse primer (specific part) can be from 4-8 bases long and the combination of two primers will still cover all of the sequence. This is in contrast to PCR methods with one specific primer, where the primer should always be as long as possible. One significant advantage of this freedom of design is that when discriminating between two miRNAs with a single base mismatch, it is easier to design primers with the 3' end close to the mismatch position, which is optimal for mismatch discrimination [27]. In agreement with this, miR-specific qPCR efficiently discriminates between related miRNAs (http://www.exiqon.com, this study).

Another indication of the robustness of miR-specific qPCR is that the PCR can be performed in different master mixes both with LNA and with DNA primers (this study).

Of the 18 assays designed for the present study, 17 worked well in qPCR, which is a success rate of 94% for primer design. For the failed primer set the forward and the reverse primers were able to form primer dimers and redesign of the primers solved this problem. By taking primer dimer formation into account it may be possible to reach even higher design success rates for DNA primers. In contrast, the success rate of LNA-spiked primers is 70% when dimer formation is ignored and 80% when accounting for putative primer dimer formation [15]. Although the primer design data set for both DNA and LNA-spiked primers are limited, the difference suggests that DNA primers may be easier to design than LNA-spiked primers in agreement with that the design rules for LNA-spiked primers are complex and slight variations in LNA number, position and sequence context can yield different results [28].

## Conclusions

In conclusion, miR-specific qPCR is a useful method for miRNA detection and the present study demonstrates that the use of DNA primers without LNA gives high PCR efficiencies that allow for precise quantification of the target.

## Methods

### Total RNA preparation

Uterus samples from 40 sows at 30-32 days of gestation were immediately snap-frozen in liquid nitrogen and stored at -80°C until use. Total RNA was extracted with TRIzol^® ^reagent (Invitrogen).

Other pig tissue samples were collected from a 3-months old Danish production pig, except for the ovary sample that was collected from a 6-months old pig. The samples were immediately snap-frozen in liquid nitrogen and stored at -80°C until use. Total RNA was extracted with TRI Reagent^® ^(Molecular Research Centre, Inc.) following the manufacturer's recommendations.

Uterus samples were obtained from Spanish pigs raised according to the European animal experimentation ethics law approved by the Ethical and Care Committee at IRTA. The rest of the tissues originated from Danish pigs raised under production conditions according to Danish standards for animal husbandry. Since the Danish animals were not subjected to experimental procedures, ethical approval was not required.

RNA quality was examined on an Agilent 2100 Bioanalyzer with the RNA 6000 Nano Kits (Agilent, Germany) or by visual inspection of the 28S/18S ribosomal bands in an agarose gel. RNA quantity was measured on a Nanodrop 1000 Spectrophotometer (Thermo Scientific, USA).

### cDNA synthesis

Total RNA was used for cDNA synthesis essentially as described [15]. Briefly, 100 ng of RNA in a final volume of 10 μl including 1 μl of 10x poly(A) polymerase buffer, 0.1 mM of ATP, 1 μM of RT-primer, 0.1 mM of each deoxynucleotide (dATP, dCTP, dGTP and dTTP), 100 units of MuLV reverse transcriptase (New England Biolabs, USA) and 1 unit of poly(A) polymerase (New England Biolabs, USA) was incubated at 42°C for 1 hour followed by enzyme inactivation at 95°C for 5 minutes. The sequence of the RT-primer was 5'-CAGGTCCAGTTTTTTTTTTTTTTTVN, where V is A, C and G and N is A, C, G and T. The primer was purchased from TAG Copenhagen (Denmark).

For the microRNA LNA™ PCR kit from Exiqon (Denmark) cDNA synthesis was done according to the manufacturer's instructions.

### Design of PCR primers and synthetic templates

All DNA PCR primers were designed according the design rules as previously described [15] except that no LNAs were spiked into the primers. Instead, Tm was optimized to 59°C by adjusting the tail length of the primers. Tm was calculated according to the nearest-neighbor model [29]. Special attention was taken to design the 3' end of the primers according to the following rules:

1. Discard all A's from the 3' end of the miRNA sequence.

2. Choose the longest possible forward primer (12 to 18 bases long) that leaves at least four bases at the 3' end of the miRNA for design of the reverse primer.

3. If possible, the last five bases at the 3' end of the forward primer should include 2-3 A or T residues.

4. If possible, the three last bases at the 3' end of the forward primer should include 1-2 A or T residues.

5. If possible, the two last bases at the 3' end of the forward primer should include 1 A or T residue.

6. If the Tm of the forward primer is below 59°C add the following bases: G, A, C, G, C at the 5' end one at a time and calculate the Tm. Choose the shortest of these primers that has a Tm = 59°C. (E.g. longest possible primer is: CGCAGN_18_, where N_18 _are 18 miR-specific bases and CGCAG is a tail sequence that is not complementary to the miR).

7. If the Tm of the forward primer is above 59°C remove bases from the 5' end one at a time and calculate the Tm. Choose the longest of these primers that has a Tm = 59°C.

8. Choose the longest possible reverse primer (4 to 8 bases long) that is not complementary to the 3' end of the forward primer.

9. Choose the reverse primer with the best 3' end according to steps 3-5.

10. Add 15 T's at the 5' end of the reverse primer.

11. If the Tm of the reverse primer is below 59°C add the following bases at the 5' end one at a time and calculate the Tm: G, A, C, C, T, G, G, A, C. Choose the shortest of these primers that has a Tm = 59°C. (E.g. longest possible primer is: CAGGTCCAGT_15_N_8_, where N_8 _are 8 miR-specific bases, T_15 _are 15 T's and CAGGTCCAG is a tail sequence complementary to the tail of the RT primer).

Synthetic templates were DNA oligonucleotides complementary to the mature sequence of the miRNAs including the RT primer sequence that is incorporated during cDNA synthesis. Sequences of primers and templates are given in Table 1. Oligonucleotides were purchased from TAG Copenhagen (Denmark) and Sigma (UK).

Primers spiked with LNA were microRNA LNA™ PCR primer sets designed by Exiqon (Denmark).

### Quantitative PCR

Quantitative PCR of biological samples was done in 10 μl total volume with 1 μl of cDNA diluted 8-10 times, 5 μl of 2x QuantiFast SYBR Green PCR master mix (Qiagen, Germany), 250 nM of each primer (Table 1) or 2 μl microRNA LNA^TM ^primer sets (Exiqon, Denmark). Standard curves with 10-fold dilutions (made with a pool of equal amounts of cDNA from the 40 uterus samples) were made for all assays to calculate qPCR efficiency.

The same PCR conditions were used for synthetic templates except that 1 μl of synthetic template in 2 ng/μl salmon sperm DNA (Sigma, USA) in TE was used instead of cDNA. 2x Brilliant III Ultra-Fast QPCR Master Mix (Agilent, USA) was used instead of QuantiFast where indicated.

Cycling conditions were 95°C for 5-10 min followed by 40 cycles of 95°C for 10-30 sec and 60°C 30-60 sec. A melting curve analysis (60°C to 99°C) was performed after the thermal profile to ensure specificity in the amplification.

QPCR of biological samples was performed on a MX3000P machine (Stratagene, USA) and reactions containing synthetic templates were performed on a Rotorcycler (Qiagen, Germany). Primers spiked with LNA were microRNA LNA™ PCR primer sets designed by Exiqon (Denmark).

### qPCR data analysis

Quantification was based on determination of the quantification cycle (Cq) and PCR efficiency was calculated from the log-linear portion of the standard curves [17].

Comparison of the efficiency of qPCR with LNA-spiked and DNA primers was done by two-sided Student's T-test for paired samples. Significance threshold was set at *P-value *< 0.05.

## Competing interests

PKB is designated as inventor of miR-specific qPCR in a patent filed by Exiqon A/S. All commercial rights to method described in the patent belong to Exiqon A/S. None of the authors have any economical interest in this company.

## Authors' contributions

PKB designed all oligonucleotides and performed and analyzed all experiments with synthetic templates. IB and SC collected biological samples, purified RNA and performed and analyzed qPCR experiments with these samples. The manuscript was written by the authors from a draft by PKB. All authors read and approved the final manuscript.
